# Supplementary material for: A comprehensive reference catalog of human skin DNA virome reveals novel viral diversity and microenvironmental influences
Source: Microbiol Spectr. 2025 Sep 30;13(11):e01178-25. doi: 10.1128/spectrum.01178-25 (PMC12584644; doi:10.1128/spectrum.01178-25)
Supplement: Supplemental figures — Fig. S1 to S6. [file spectrum.01178-25-s0001.docx]

**Supplementary Figure**


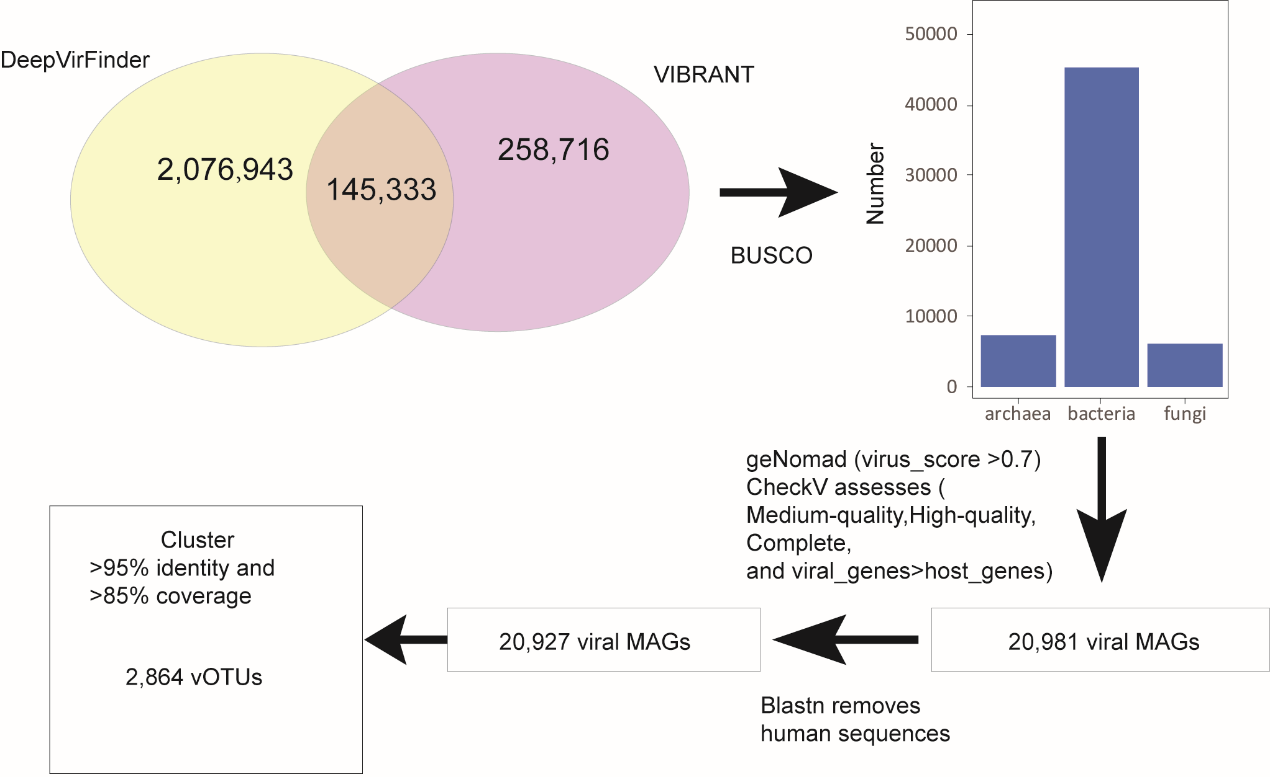


**Figure S1. An overview of the viral OTUs detection process.**


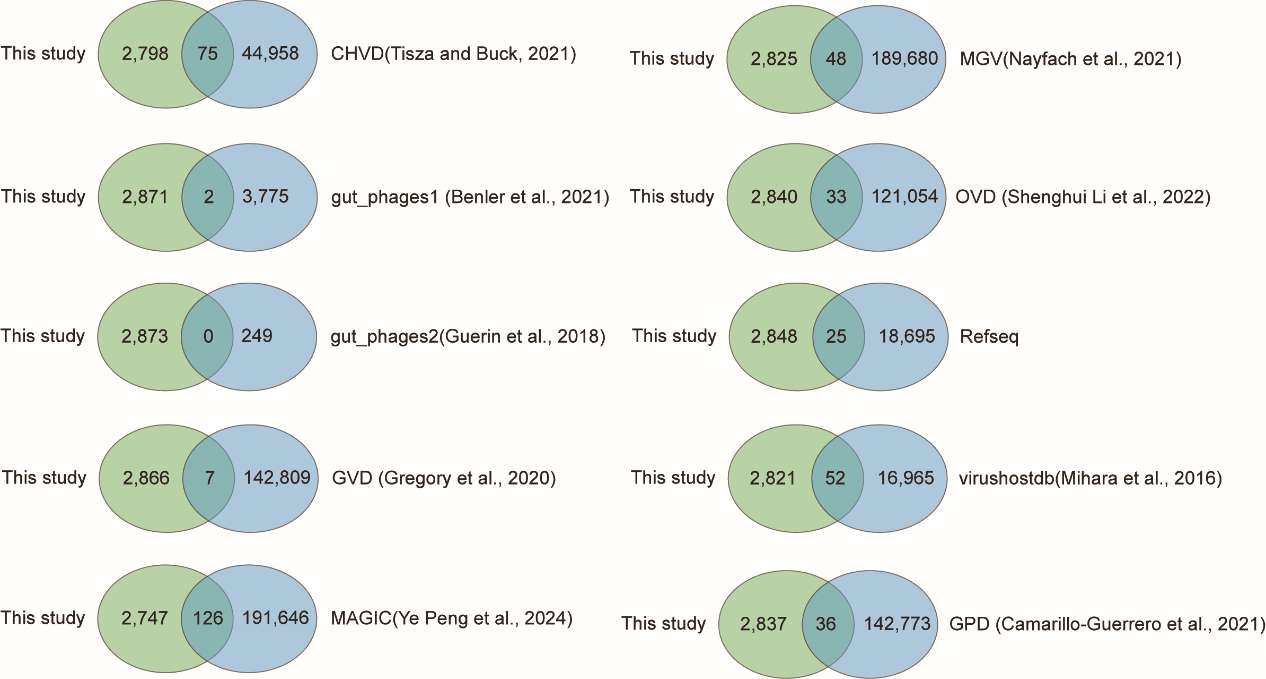


**Figure S2. Comparison of SVD with Other Viral Databases.**

**
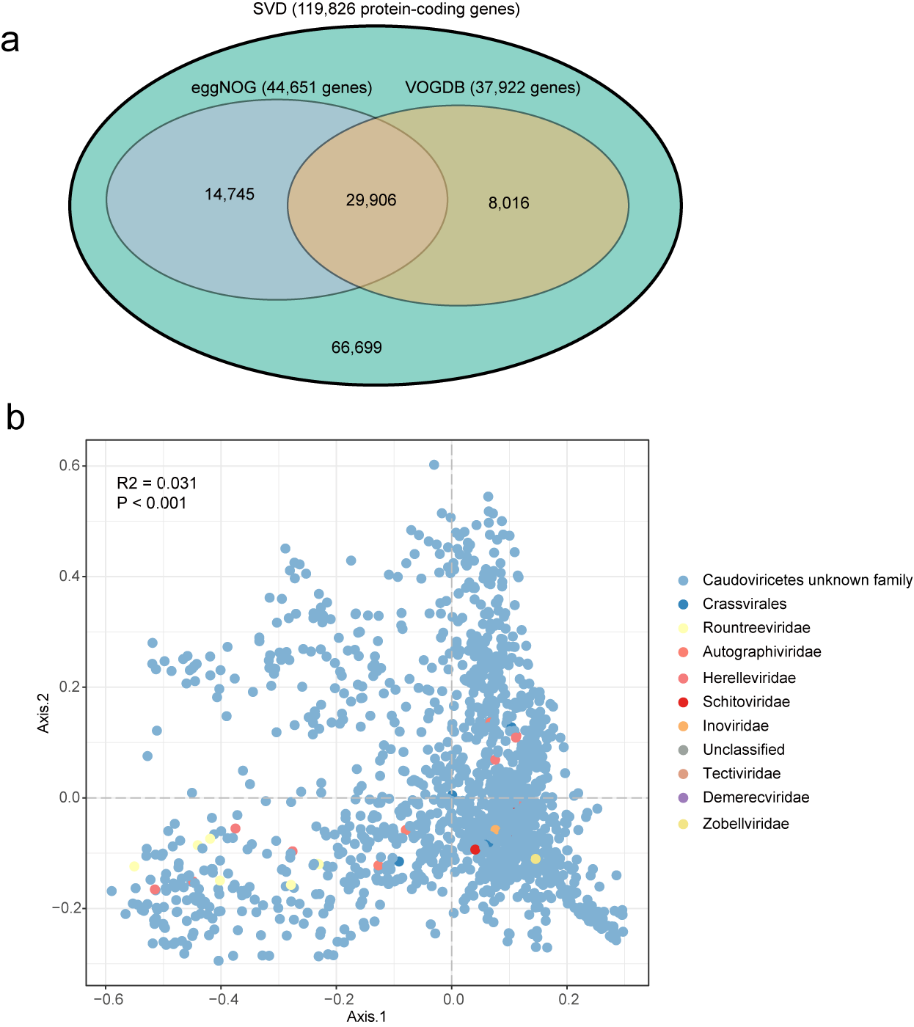
**

**Figure S3. Overview of the viral functions in SVD. a.** Proportion of identified and unidentified functions after annotation through the eggNOG database and VOGDB. **b.** PCoA demonstrates significant functional differences among viruses at different genus levels.


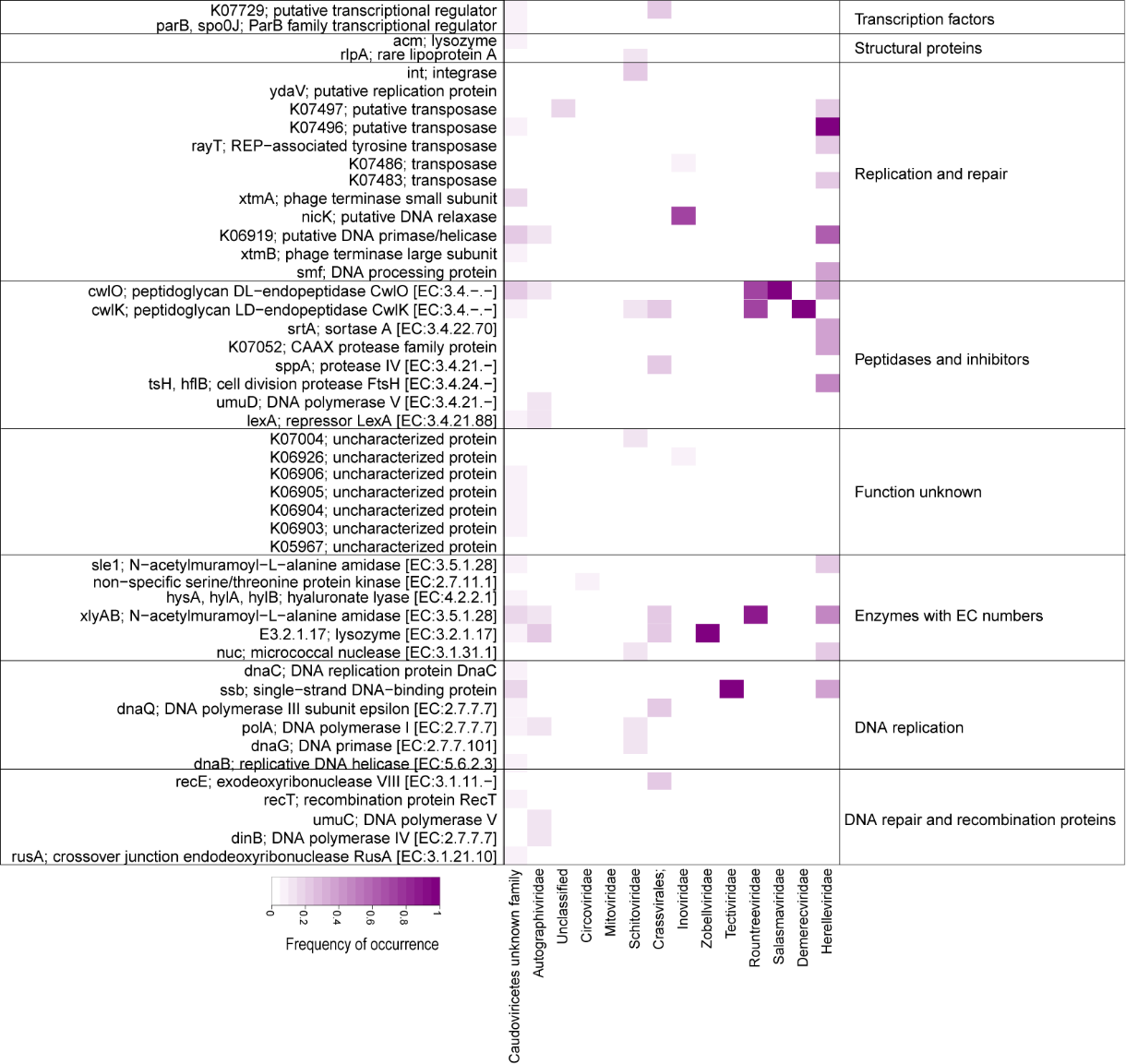


**Figure S4. Potential functional differences of skin vOTUs.** The heatmap displays the proportion of vOTUs that possess a particular function. A darker color indicates a higher number of vOTUs with that function in the genus.


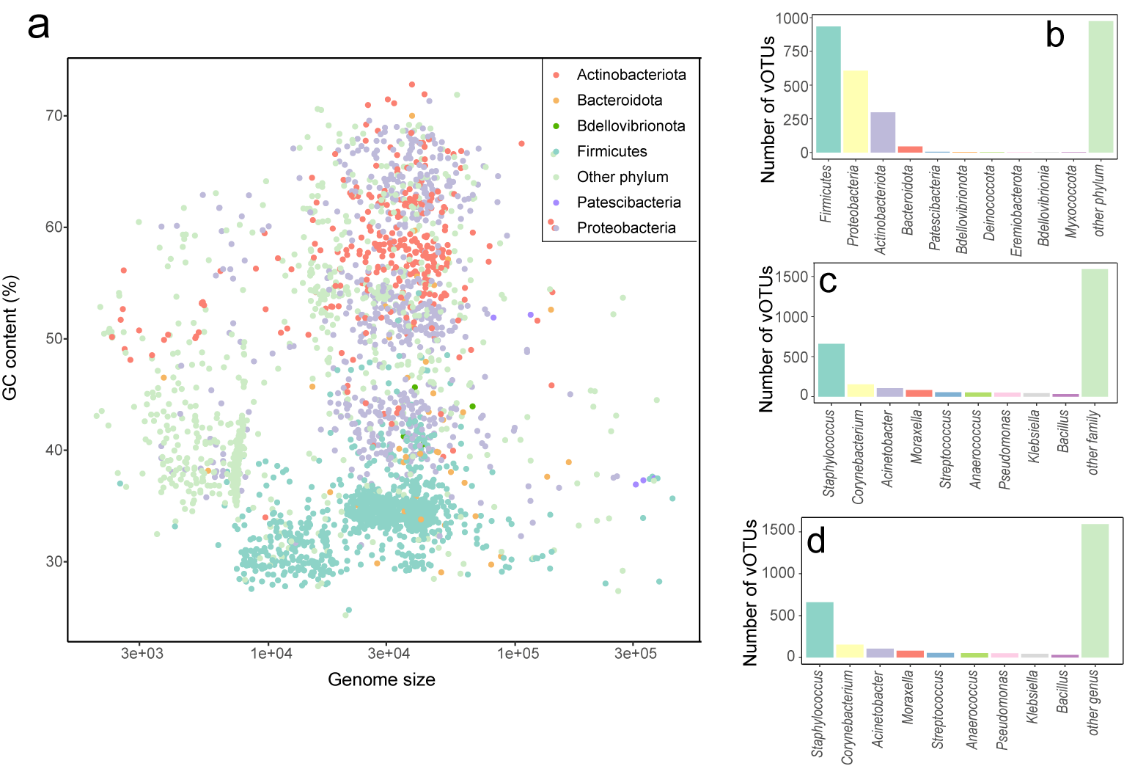


**Figure S5. Host information of reconstructed bacteriophage genomes. a.** Genome size and GC content of SVD reconstructed from a dataset of 2,760 complete metagenomes. **b-d.** Barplots showing the number of bacteriophage hosts at the phylum (b), family (c), and genus (d) levels in SVD, respectively.


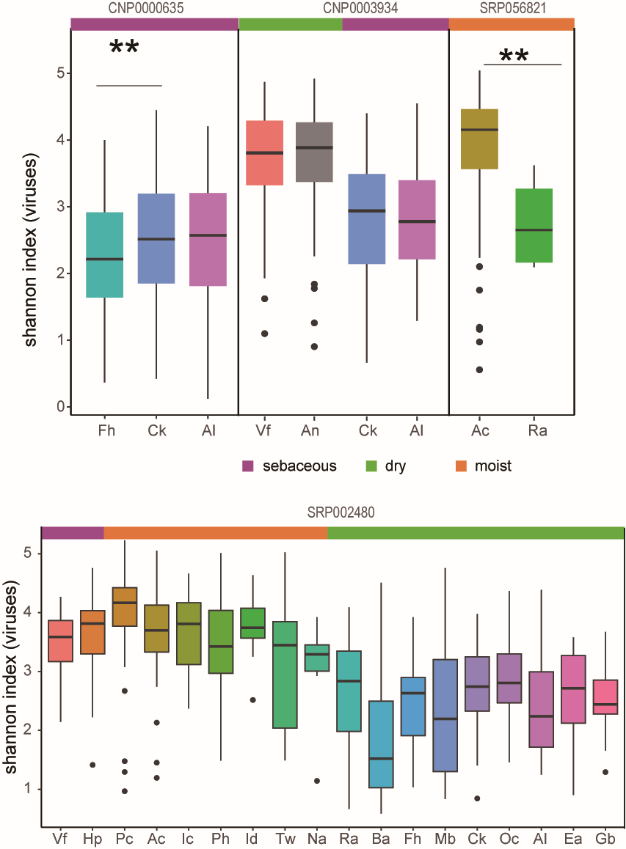


**Figure S6. Differences in viral Shannon diversity across different microenvironments and sites.** Fh: forehead, Ck: cheek, AI: Alar crease, Ea: external auditory canal, Ra: retroarticular crease, Oc: occiput, Ba: back, Mb: manubrium, Na: nare, Ac: antecubital fossa, Id: interdigital web, Pc: popliteal fossa, Ic: inguinal crease, Vf: volar forearm, Hp: hypothenar palm, Tw: toe webspace, Tn: toenail, Ph: plantar heel, Gb: Glabella.

**Supplementary Tables**

**Table S1. Size and characteristics of skin metagenomic samples in this study.**

**Table S2.** **Detailed information of the skin metagenomic samples, assembly and human reads proportion.**

**Table S3. Taxonomy, geNomad and CheckV assessment of the medium/high-quality vOTUs.**

**Table S4. The classification, clustering, CheckV evaluation, and iPHoP host prediction results of medium- and high-quality vOTUs in SVD.**

**Table S5.** **Results of the Shannon index Wilcoxon test for different sites within the same environment.**

**Table S6. Effect size of metadata variables on skin virome composition at the vOTU level.**

**Table S7. Environment-specific viruses in different cohorts.**
